# Molecular Identification and Antifungal Susceptibility Pattern of Non-*albicans Candida* Species Isolated from Vulvovaginal Candidiasis

**DOI:** 10.22034/ibj.22.1.33

**Published:** 2018-01

**Authors:** Ziba Abbasi Nejat, Shirin Farahyar, Mehraban Falahati, Mahtab Ashrafi Khozani, Aga Fateme Hosseini, Azamsadat Faiazy, Masoome Ekhtiari, Saeideh Hashemi-Hafshenjani

**Affiliations:** 1 International Campus, Iran University of Medical Sciences, Tehran, Iran; 2 Department of Medical Parasitology and Mycology, School of Medicine, Iran University of Medical Sciences, Tehran, Iran; 3 Microbial Biotechnology Research Center, School of Medicine, Iran University of Medical Sciences, Tehran, Iran; 4 Department of Biostatistics, School of Public Health, Iran University of Medical Sciences, Tehran, Iran; 5 Department of Gynecology, Sayyad Shirazi Hospital, Golestan University of Medical Sciences, Gorgan, Iran

**Keywords:** *Candida glabrata*, Vulvovaginal candidiasis, *Candida krusei*

## Abstract

**Background:**

Vulvovaginal candidiasis (VVC) is an important health problem caused by *Candida* spp. The aim of this study was molecular identification, phylogenetic analysis, and evaluation of antifungal susceptibility of non-*albicans Candida* isolates from VVC.

**Methods:**

Vaginal secretion samples were collected from 550 vaginitis patients at Sayyad Shirazi Medical and Educational Center of Gorgan (Golestan Province, Iran) from May to October 2015. Samples were analyzed using conventional mycological and molecular approaches. Clinical isolates were analyzed with specific PCR using CGL primers, and the internal transcribed spacer region and the D1-D2 domain of the large-subunit rRNA gene were amplified and sequenced. Susceptibility to amphotericin B, fluconazole, itraconazole, and clotrimazole was determined by the guidelines of the Clinical and Laboratory Standard Institute.

**Results:**

In total, 35 non-*albicans Candida* isolates were identified from VVC patients. The isolates included 27 strains of *Candida glabrata* (77.1%), 5 *Candida krusei* (*Pichia kudriavzevii*; 14.3%), 2 *Candida kefyr* (*Kluyveromyces marxianus*; 5.7%), and 1 *Candida lusitaniae* (*Clavispora lusitaniae*; 2.9%). The fungicides itraconazole and amphotericin B were effective against all species. One isolate of *C. glabrata* showed resistance to fluconazole and clotrimazole, and 26 isolates of *C. glabrata* indicated dose-dependent susceptibility to fluconazole. *C. lusitaniae* was susceptible in a dose-dependent manner to fluconazole and resistant to clotrimazole.

**Conclusions:**

Non-*albicans Candida* spp. are common agents of vulvovaginitis, and *C. glabrata* is the most common species in the tested patients.

## INTRODUCTION

The incidence of vulvovaginal candidiasis (VVC) caused by non-*albicans Candida* spp. has increased considerably[1,2]. Based on evidence, about two-thirds of women worldwide have experienced at least one episode of VVC during their lifetime[3] and some with recurrent episodes[1]. Recurrent episodes are more often caused by non-*albicans Candida* spp. against which azole antifungal agents show low effectiveness[4].

*Candida albicans* is the major cause of vulvovaginitis, and *Candida glabrata* and *Candida tropicalis* appear to be the most common non-*albicans Candida* species involved in this disease[2,4-6]. Identification of many species of *Candida* by traditional methods is a challenge and is sometimes imprecise, particularly for uncommon microorganisms. Sequence analysis of the internal transcribed spacer (ITS) region of the rRNA gene and the D1-D2 domain of the large-subunit rRNA gene as well as PCR-RFLP on the ITS region have been used extensively for identification of fungal pathogens[7-11].

Minimal inhibitory concentrations (MIC) of azoles against some strains of non-*albicans Candida* species are high, due to intrinsic resistance[12]. The antifungal susceptibility of *Candida* spp. causing VVC varies[12,13], and reliable identification and assessment of drug sensitivity of *Candida* vaginal isolates are of value in determining proper treatment. The aim of this study was molecular identification, phylogenetic analysis, and evaluation of antifungal susceptibility of non-*albicans Candida* isolates causing VVC.

## MATERIALS AND METHODS

### Patients and specimens collection

This study was conducted on 550 non-pregnant vaginitis patients referred to Sayyad Shirazi Medical and Educational Center of Gorgan (Golestan, Iran) from May to October 2015. Non-pregnant patients were entered to the study by a simple random sampling method. A questionnaire was completed for each patient about their age, the medical condition (recent antibiotic or antifungal therapy, urinary tract infections, immunodeficiency, diabetes, experiencing at least one episode or recurrent episodes of VVC), and other conditions. Specimens were obtained from vaginal mucosal discharge with a sterile cotton swab. The research protocol was approved by the Ethics Committee of Iran University of Medical Sciences (Tehran, Iran), under Ethics Committee number 93-04-198-25289.

### Yeast identification

Microscopic examination was carried out to distinguish yeast forms or pseudohyphae. All samples were cultured on CHROMagar Candida (CHROMagar, France) for identification of mixed infections of *Candida* spp.[14]. The isolates were identified by carbohydrate assimilation method using API 20C AUX system (Biomérieux, France)[15].

### DNA extraction

A single colony of each clinical isolate from CHROMagar Candida was subcultured on yeast extract peptone dextrose agar and incubated at 37 °C for 24-48 h. Genomic DNA was extracted from yeast cultures using the Qiagen DNA tissue kit (Germany). The extracted DNA was stored at -20 °C for further use.

### Specific PCR

All clinical isolates with mauve, pink, or white colonies on CHROMagar Candida as well as *C. glabrata* CBS 138, as the reference strain, were analyzed by *C. glabrata*-specific PCR with CGL1-(5´-TTA TCA CAC GAC TCG ACA CT-3´) and CGL2- (5´-CCC ACA TAC TGA TAT GGC CTA CAA-3´)[7] primers. The PCR thermal cycles were as follows: an initial denaturation at 96 °C for 5 min followed by 40 cycles of 30 s at 94 °C, 30 s at annealing temperature of 58 °C and 30 s at 72 °C. A final extension of 15 min at 72 °C was included at the end of PCR cycles.

### Amplification and sequencing of ITS and D1-D2 regions

The universal primers ITS1 (5´-TCC GTA GGT GAA CCT GCG G -3´) and ITS4 (5´-TCC TCC GCT TAT TGA TAT GC-3´)[7] were used to amplify the ITS1-5.8S-ITS2 region (annealing temperature 56 °C). Also, D1-D2 domain of 26S ribosomal RNA was amplified with NL1 (5´-GCA TAT CAA TAA GCG GAG GAA AAG-3´) and NL4 (5´- GGT CCG TGT TTC AAG ACG G-3´)[16] primers by the following profile: 98 °C (5 min), 35 cycles of 98 °C (30 s), annealing temperature 60 °C (30 s), and 72 °C (30 s), followed by a final extension of 72 °C (5 min). The PCR products were sequenced by Macrogen (Korea). The resulting sequences were analyzed and compared with the reference data available from the GenBank database using the BLAST sequence search tool (http://www.ncbi.nlm.nih.gov/BLAST), and the results were submitted to the GenBank.

### Phylogenetic analysis

The sequencing results of the D1-D2 and the ITS domains were analyzed and compared with the reference strains by neighbor-joining method using MEGA 7 (TreeView software).

### Antifungal drug susceptibility testing

Tests of susceptibility to amphotericin B, fluconazole, clotrimazole, and itraconazole (Sigma, Germany) were conducted according to the Clinical and Laboratory Standards Institute (CLSI) guidelines (document M27-S3 and S4)[17,18]. *C. glabrata* CBS 138 was used as the reference strain, and all tests were duplicated.

## RESULTS

### Patients

A total of 550 vaginal specimens of non-pregnant vaginitis patients were studied. Individuals with conditions such as infection by *Trichomonas vaginalis*, *Mycoplasma urealyticum*, or *Chlamydia*, as well as bacterial vaginosis or vulval skin disease were excluded from the study. In addition, 122 (22.2%) non-pregnant vaginitis patients showed VVC, and *C. albicans* isolates were identified in 87 (71.3%) VVC patients (data not shown). Non-*albicans Candida* isolates were found in 35 (28.7%) VVC patients aged 19-39 years from Gorgan (Table 1). All patients were negative for diabetes, immunodeficiencies, or any chronic disease and were not taken any antifungal treatment.

**Table 1:** Non-*albicans Candida* isolates and age distribution of vulvovaginal candidiasis patients

Ages (y)	*Candida* spp.			

	*C. glabrata*	*C. krusei*	*C. kefyr*	*C. lusitaniae*
<20	6	3	1	-
20-29	10	2	1	-
30-39	11	-	-	1

### Yeast isolates

Thirty-five isolates of non-*albicans Candida* were obtained from 550 vulvovaginitis patients: 27 *C. glabrata* (77.1%), 5 *C. krusei* (*Pichia kudriavzevii*; 14.3%), 2 *C. kefyr* (*Kluyveromyces marxianus*; 5.7%), and 1 *Candida lusitaniae* (*Clavispora lusitaniae*; 2.9%) (Table 1).

### Amplification with specific primers

**Fig.1:**
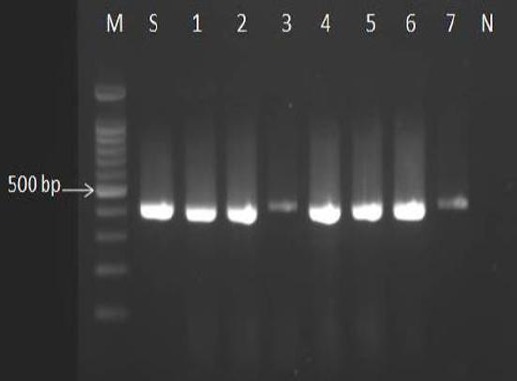
The genomic DNA of clinical isolates of *Candida glabrata* and *C. glabrata* CBS 138 were analyzed with PCR using CGL1/2 specific primers, and a 423-bp fragment produced. Isolates 1, 2, 3, 4, 5, 6, and 7, *C. glabrata*; S, *C. glabrata* CBS 138 (as standard); M, marker 100 bp; N, negative control.

The clinical isolates with mauve, pink, or white colonies on CHROMagar Candida and *C. glabrata* CBS 138 were analyzed with CGL1/2 specific primers, and the presence of the 423-bp fragment amplified with these primers confirmed those isolates identical to *C. glabrata* (Fig. 1).

### PCR amplification and sequencing of ITS region and D1-D2 domain

**Fig.2:**
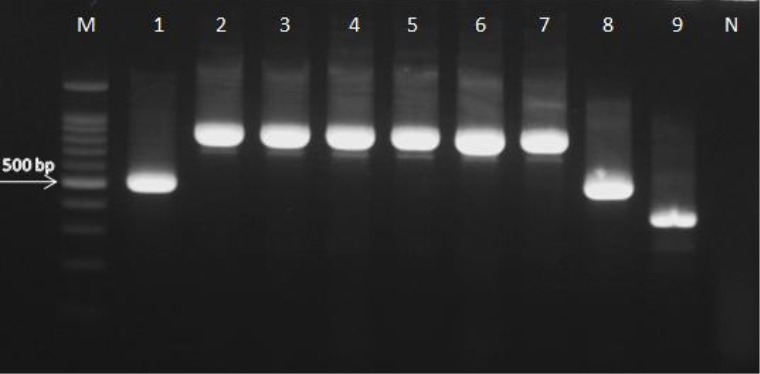
Amplification of genomic DNA from clinical isolates using ITS1 and ITS4 primers. Isolates 1 and 8, *C. krusei* (500 bp); isolates 2, 3, 4, 5, and 6, *Candida glabrata* producing an ~879-bp fragment; isolate 7, *C. glabrata* CBS 138 (as standard); isolate 9, *Candida lusitaniae* producing a ~370-bp fragment; M, marker 100 bp; N, negative control.

**Fig.3:**
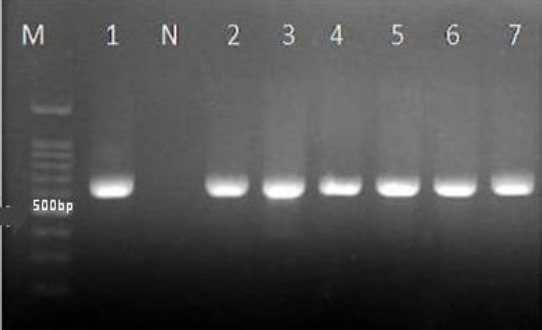
The D1-D2 region of clinical isolates amplified with NL1 and NL4 primers, yielded fragments ~600 bp. Isolate 1, *C. glabrata* CBS 138 (as standard); isolates 2, 3, 4, 5, 6, and 7,: *Candida glabrata*; M, marker 100 bp; N, negative control.

PCR amplification of all clinical isolates with ITS1 and ITS4 primers yielded the fragments of 350-880 bp. The ITS fragments of *C. glabrata* were ~500 to ~879 bp (Fig. 2)[8,10], while *C. krusei*, *C. kefyr*, and *C. lusitaniae* yielded the fragments of ~500, ~720, and ~370 bp, respectively (Fig. 2)[8]. The ITS fragments of three clinical *C. glabrata* isolates showing ~500 and ~600 bp were compared to the reference data in the GenBank database using the BLAST. Three *C. glabrata* isolates showed partial sequences of ITS region (~500 and ~600 bp), while the complete sequences of ITS region of *C. glabrata* was ~879 bp, and the partial sequences and complete sequences were submitted to the GenBank (Table 2). The D1-D2 region of the large-subunit rRNA gene amplified with NL1 and NL4 primers yielded the fragments of ~600 bp (Fig. 3). The ITS and D1-D2 region sequences of non-*albicans Candida* clinical isolates were compared to the reference data in the GenBank database using BLAST (http://www.ncbi.nlm.nih.gov/BLAST). All clinical isolates were correctly determined to species level. The sequences were submitted to the GenBank under accession numbers KU845721, KU904424-26, KU992386-95, KX008737-53, and KX016018-21 (Table 2).

**Table 2:** Accession numbers of clinical isolates

Clinical isolates	Accession no.
*C. glabrata*	KU845721
	KU904426
	KU992388
	KU992389
	KU992390
	KU992391
	KU992392
	KU992393
	KU992394
	KU992395
	KX008737
	KX008738
	KX008739
	KX008740
	KX008741
	KX008744
	KX008745
	KX008748
	KX008749
	KX008750
	KX008751
	KX008752
	KX008753
	KX016018
	KX016019
	KX016020
	KX016021
*Pichia kudriavzevii (Candida krusei)*	KU904424
	KU992387
	KX008742
	KX008743
	KX008746
*Kluyveromyces marxianus(Candida kefyr)*	KU992386
	KX008747
*Clavispora lusitaniae (Candida lusitaniae)*	KU904425

### Phylogenetic trees

**Fig.4:**
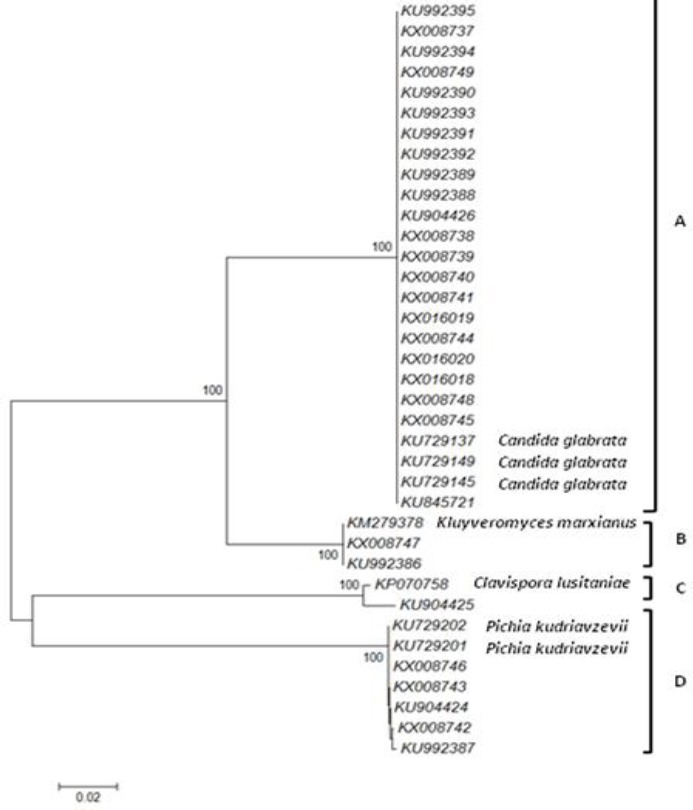
Molecular phylogenetic analysis using Neighbor-Joining method with sequences of D1-D2 domain. The evolutionary history was inferred using the Neighbor-Joining method. The percentage of replicate trees in which the associated taxa clustered together in the bootstrap test (1000 replicates) is shown next to the branches. Bootstrap values greater than 50% from 1000 replicates are indicated at the nodes. The tree is drawn to scale, with branch lengths in the same units as those of the evolutionary distances used to infer the phylogenetic tree. Evolutionary analyses were conducted in MEGA7. (A) Accession numbers of *Candida glabrata* isolated in this study and reference strains (KU729149, KU729145, and KU729137), (B) accession numbers of *Kluyveromyces marxianus* (*Candida kefyr*) isolated in this study and reference strain (KM279378), (C) Accession number of *Clavispora lusitaniae* (*Candida lusitaniae*) isolated in this study and reference strain (KP070758), (D) accession numbers of *Pichia kudriavzevii* (*Candida krusei*) isolated in this study (Table 1) and reference strains (KU729202 and KU72920).

**Fig.5:**
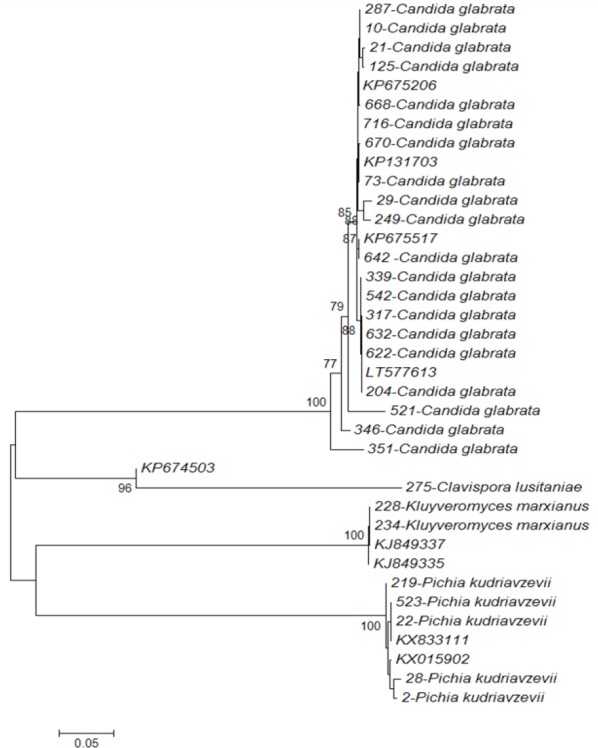
Molecular phylogenetic analysis using Neighbor-Joining method with sequences of ITS region. The evolutionary history was inferred using the Neighbor-Joining method. The percentage of replicate trees in which the associated taxa clustered together in the bootstrap test (1000 replicates) is shown next to the branches. Bootstrap values greater than 70% from 1000 replicates are indicated at the nodes. The tree is drawn to scale, with branch lengths in the same units as those of the evolutionary distances used to infer the phylogenetic tree. Evolutionary analyses were conducted in MEGA7.

The sequences of D1-D2 region were aligned for phylogenetic analysis. All *C. glabrata* strains showed 100% identity with KU729149, KU729145, and KU729137 reference strains. *C. krusei* (*Pichia kudriavzevii*) indicated 100% similarity to KU729202 and KU729201 reference strains. *C. kefyr* (*Kluyveromyces marxianus*) and *C. lusitaniae* (*Clavispora lusitaniae*) displayed 100% identity with KM279378 and KP070758 reference strains, respectively (Fig 4). Phylogenetic analysis of sequences corresponding to the ITS region demonstrated that all strains of the species were identical to the reference strains. *C. glabrata* strains showed similarity to KP675206, KP131703, KP675517, and LT577613 but *C. krusei* (*Pichia kudriavzevii*) to KX833111 and KX015902 reference strains. *C. kefyr* (*Kluyveromyces marxianus*) indicated identity with KJ849337 and KJ849335 reference strains, while *C. lusitaniae* (*Clavispora lusitaniae*) showed similarity to KP674503 reference strain (Fig. 5).

### Antifungal drug susceptibility

Results of susceptibility testing of the 35 non-*albicans Candida* isolates showed one isolate of *C. glabrata* to be resistant to fluconazole (MIC ≥ 64 µg/ml) and clotrimazole (MIC ≥ 4 µg/ml), and 26 isolates of *C. glabrata* were susceptible to fluconazole (MIC ≤ 32 µg/ml) in a dose-dependent manner[18]. Single clinical isolate of *C. lusitaniae* showed dose-dependent susceptibility to fluconazole (MIC = 16-32 µg/ml) and resistant to clotrimazole (MIC = 2 µg/ml)[17]. The MICs for one isolate of *C. krusei* were as follows: fluconazole ≤ 32 µg/ml and clotrimazole = 2 µg/ml. Because the clinical isolates of *C. krusei* showed intrinsic resistant to fluconazole, and their MICs should not be interpreted using this scale; therefore, breakpoint was not provided by CLSI document M27-S4[18]. Itraconazole and amphotericin B were active against all of the isolates (Table 3).

**Table 3:** Drug treatment susceptibility of *Candida* spp. isolated from vulvovaginal candidiasis patients

Clinical isolates	Fluconazole			Itraconazole			Clotrimazole			Amphotericin B		
			
	S	R	S-DD	S	R	S-DD	S	R	S-DD	S	R	S-DD
		
	n			n			n			n		
*C. glabrata* (n = 27)	0	1	26	27	0	0	26	1	0	27	0	0
*C. krusei* (n = 5)	--*	--	--	5	0	0	4	1	0	5	0	0
*C. kefyr* (n = 2)	2	0	0	2	0	0	2	0	0	2	0	0
*C. lusitaniae* (n = 1)	0	0	1	1	0	0	0	1	0	1	0	0

S, sensitive; R, resistant; S-DD, susceptible, dose-dependent;

* Because of the clinical isolates of *C. krusei* showed intrinsically resistant to fluconazole; therefore, breakpoint is not provided by Clinical and Laboratory Standards Institute document M27-S4.

## DISCUSSION

This study revealed that non-*albicans Candida* spp., as important agents, are commonly associated with vulvovaginitis; *C. glabrata* is the second in rate of occurrence after *C. albicans*. Other investigations have found that *C. glabrata* and *C*. *albicans* to be the most common species isolated from VVC patients[13,19,20]. The overall proportion of non-*albicans* infection in vaginitis has been reported to be high[2]. *C. glabrata* and *C. tropicalis* have also been found in the normal vaginal flora of women in China[21]. Infections caused by less common yeasts have been increasingly observed[22], and identification of a variety of medically important yeast species by traditional approaches may be challenging. Molecular methods can improve discrimination of uncommon clinical isolates and closely related yeast species such as those in *Candida* complexes. Molecular diagnostics are also useful in carrying out large epidemiological studies of pathogenic yeasts. In this study, conventional methods and specific PCR with CGL primers were used for identification of *C. glabrata*. Sequencing the ITS and D1-D2 regions has proven to be a feasible method for the reliable identification of clinically important yeasts, principally the *C. glabrata* complex (*C. glabrata*, *C. bracarensis*, and *C. nivariensis*)[22,23] and the *C. parapsilosis* complex (*C. parapsilosis*, *C. ortho-psilosis*, and *C. metapsilosis*)[24]. Richter *et al*.[13] reported that 173 of 593 yeast isolates from vaginitis patients were non-*albicans Candida* spp., and that *C. albicans* was the most frequent cause of vaginal candidiasis, followed by *C. glabrata*, *C*. *parapsilosis*, *C. krusei, Saccharomyces cerevisiae*, *C. tropicalis*, and *C. lusitaniae*[13]. Vijaya *et al*.[25] showed that *C. tropicalis* is the major non-*albicans* species of *Candida* associated with vaginal candidiasis. Other studies in Iran introduced *C. glabrata* as the most important non-*albicans* species in vaginal candidiasis patients[19,20]. Shi *et al*.[26] demonstrated that *C. albicans* is the main cause of vaginal candidiasis, followed by *C. glabrata*, *C. tropicalis* and *C. parapsilosis* in China. In the current study, *C. glabrata* was the most common species of non-*albicans Candida*. *Candida* spp., especially *C. glabrata* and *C. albicans*, represent a primary source of infection leading to bloodstream infections and to morbidity and mortality in severely affected and immune-compromised individuals[12,27-29]. Species *C. krusei* and *C. glabrata* have been indicated. To be resistant or to have low susceptibility to azole drugs[4,12]. *C. lusitaniae* has also been shown to have resistance to amphotericin B, caspofungin, and azoles[30]. A study in Japan revealed that one of the 19 *C. glabrata* clinical isolates of VVC patients showed resistance to fluconazole, and this isolate demonstrated cross-resistance to other antimycotic drugs tested[31]. Another study displayed that non-*albicans* isolates, particularly *C. glabrata* strains, were susceptible in a dose-dependent manner and were resistant to fluconazole[13]. Kalkanci *et al*.[32] suggested that *C. glabrata* was the most vaginal isolate of non-*albicans Candida*, and 3 of 81 (3.7%) *C. glabrata* isolates were resistant to ketoconazole, and only one *C. glabrata* was fluconazole resistant. Also, five *C. glabrata* isolates showed susceptibility to fluconazole in a dose-dependent manner. A previous study indicated that nystatin was an appropriate option instead of imidazoles[33]. An investigation from Iran found that clinical isolates of *Candida* spp. were susceptible to clotrimazole, miconazole, and nystatin[19]. Razzaghi-Abyaneh *et al*.[34] indicated that itraconazole was the most effective antimycotic drug for *C. krusei*, *C. glabrata*, and *C. guilliermondii* isolates of superficial candidiasis in Iran. In the current study, 26 isolates of *C. glabrata* were susceptible dose-dependent to fluconazole, and one isolate was resistant to fluconazole and clotrimazole. In addition, one isolate of *C. lusitaniae* was susceptible to fluconazole in a dose-dependent manner and resistance to clotrimazole[35,36].

The phylogenetic analyses of the D1-D2 and the ITS domains indicated that clinical isolates of vaginal candidiasis are genetically similar to reference *Candida* species. The phylogenetic analyses of the D1-D2 domain revealed that all *C. glabrata* isolates had 100% similarity to KU729149 (ATCC 90030), KU729145 (ATCC 66032), and KU729137 (ATCC 2001) reference strains. Clinical isolates of *C. krusei* (*Pichia kudriavzevii*) showed 100% identity with KU729202 (ATCC 34135) and KU729201 (ATCC 14243) reference strains. *C. kefyr* (*Kluyveromyces marxianus*) and *C. lusitaniae* (*Clavispora lusitaniae*) were similar to KM279378 (isolate U-MF11) and KP070758 (isolate 0Q10) reference strains, respectively. Based on the phylogenetic analyses of the ITS region, *C. glabrata* clinical isolates showed similarity to KP675206 (strain m36b), KP131703 (CNRMA6.53 isolate ISHAM-ITS_ID MITS649), KP675517 (strain M310B), and LT577613 (strain IQBasrah28) reference strains in the GenBank databases. *C. krusei* (*Pichia kudriavzevii*) showed similarity to KX833111 (strain DMic 165166) and KX015902 reference strains. *C. kefyr* (*Kluyveromyces marxianus*) amplified sequences matched completely with the corresponding sequences of the KJ849337 (strain ZT-Kma.4) and KJ849335 reference strains. *C. lusitaniae* (*Clavispora lusitaniae*) indicated 96% identity with KP674503 (strain B157B) reference strain. The phylogenetic trees were created using the sequences of different *Candida* clinical isolates and showed the formation of separate branches for each species.

Sequencing of the ITS region and D1-D2 domain appears to be the most effective method for identification of *Candida* spp. The phylogenetic trees based on sequences of D1-D2 and ITS domains showed similarity of *Candida* spp. to closely related reference species. Results suggested that amphotericin B and itraconazole retain good clinical effectiveness. Accurate identification and assessment of susceptibility of *Candida* spp. isolates are critical to treatment management, since some strains showed varying degrees of resistance to antifungal drugs.
